# Deciphering Normal Blood Gene Expression Variation—The NOWAC Postgenome Study

**DOI:** 10.1371/journal.pgen.1000873

**Published:** 2010-03-12

**Authors:** Vanessa Dumeaux, Karina S. Olsen, Gregory Nuel, Ruth H. Paulssen, Anne-Lise Børresen-Dale, Eiliv Lund

**Affiliations:** 1 Institute of Community Medicine, University of Tromsø, Tromsø, Norway; 2 Department of Applied Mathematics, University of Paris Descartes, Paris, France; 3 Institute of Clinical Medicine, Faculty of Medicine, University of Tromsø, Tromsø, Norway; 4 Department of Genetics, Institute for Cancer Research, Oslo University Hospital Radiumhospitalet, Oslo, Norway; 5 Institute of Clinical Medicine, University of Oslo, Oslo, Norway; University of Geneva Medical School, Switzerland

## Abstract

There is growing evidence that gene expression profiling of peripheral blood cells is a valuable tool for assessing gene signatures related to exposure, drug-response, or disease. However, the true promise of this approach can not be estimated until the scientific community has robust baseline data describing variation in gene expression patterns in normal individuals. Using a large representative sample set of postmenopausal women (N = 286) in the Norwegian Women and Cancer (NOWAC) postgenome study, we investigated variability of whole blood gene expression in the general population. In particular, we examined changes in blood gene expression caused by technical variability, normal inter-individual differences, and exposure variables at proportions and levels relevant to real-life situations. We observe that the overall changes in gene expression are subtle, implying the need for careful analytic approaches of the data. In particular, technical variability may not be ignored and subsequent adjustments must be considered in any analysis. Many new candidate genes were identified that are differentially expressed according to inter-individual (i.e. fasting, BMI) and exposure (i.e. smoking) factors, thus establishing that these effects are mirrored in blood. By focusing on the biological implications instead of directly comparing gene lists from several related studies in the literature, our analytic approach was able to identify significant similarities and effects consistent across these reports. This establishes the feasibility of blood gene expression profiling, if they are predicated upon careful experimental design and analysis in order to minimize confounding signals, artifacts of sample preparation and processing, and inter-individual differences.

## Author Summary

As a major defence and transport system, blood cells are capable of adjusting gene expression in response to various clinical, biochemical, and pathological conditions. Here, we expand our understanding about the nature and extent of variation in gene expression from blood among healthy individuals. Using a large representative sample of postmenopausal women (N = 286) in the Norwegian Women and Cancer (NOWAC) postgenome study, we investigated blood gene expression changes due to normal inter-individuality (age, body mass index, fasting status), and exposure variables (smoking, hormone therapy, and medication use) at proportions and levels found in real life situations. Host genes were found to vary by inter-individual (i.e. fasting, BMI) and exposure (i.e. smoking) factors, and these gene lists may be used as a basis for further hypothesis development. Our study also establishes the feasibility of blood gene expression profiling for disease prediction, diagnosis, or prognosis, but underscores the necessity of care in study design and analysis to account for inter-individual differences and confounding signals.

## Introduction

There is growing evidence that transcriptome analysis of peripheral blood cells is a valuable tool for determining signatures related to disease [1]–[5] and drug-response [6]. Differences in blood gene expression may also reflect the effects of a particular exposure, such as smoking [7], metal fumes [8], or ionizing radiation [9]. In our previous research, we studied gene expression profiles from whole blood related to hormone therapy (HT) use in postmenopausal women [10] and identified specific challenges raised by inter-individual variability when isolating signals associated with defined exposure levels. Although blood gene expression profiling promises molecular-level insight into disease mechanisms, there remains a lack of baseline data describing the nature and extent of variability in blood gene expression in the general population. Characterizations of this variation and the underlying factors that most influence gene expression amongst healthy individuals will play an important role in the feasibility, design and analysis of future blood-based studies investigating biomarkers for exposure, disease progression, diagnosis or prognosis [11].

Several studies [12]–[18] have reported that technical variables such as collection, transportation, storage of blood samples, RNA isolation method and choice of microarray platform, in addition to biological effects, can influence gene expression profiles. These technical factors associated with the processing and preparation of human blood and subsequent microarray hybridization represent significant challenges in the analysis of variability.

Furthermore, a few previous studies have used microarrays to analyze blood from healthy volunteers and found that inter-individual sample variation was associated with sex [18], age [13],[18], the time of day the sample was taken [18],[19], and the proportion of the different cell populations comprising the blood sample [13],[18],[20]. However to date, all such studies have focused on gene expression profiles generated from a small set of samples not representative of the general population using different blood cell subtypes. For several reasons including the small sample sizes, these studies have been restricted to the analysis of a small number of variables simultaneously, thus ignoring possible interaction and confounder effects.

Finally, an understanding of these causes of variability would represent a significant step forward in the identification and evaluation of the disease and disease risk biomarkers. Most if not all genes are involved in molecular pathways that provide mechanistic insight in response to exposure or disease development. Pathway depictions are usually simplified, ignoring interactions with other pathways, and we often have incomplete knowledge about the specific interplay of the many elements in almost any particular system.

Using a large representative sample set of postmenopausal women in the Norwegian Women and Cancer (NOWAC) postgenome study [21],[22] processed via a standardized blood collection procedure and via an experimentally validated microarray platform [23], we investigate here the baseline variability of whole blood gene expression profiles. This represents the first comprehensive cross-sectional analysis of blood gene expression changes related to multiple inter-individual and exposure variables, and opens the new research discipline of systems epidemiology [24]. In this setting, we investigated blood gene expression changes due to technical variability, normal inter-individuality, and exposure variables at proportions and levels relevant to real life situations, and establish that these effects are mirrored in the blood transcriptome.

## Results

### Study design

#### Population characteristics

Characteristics of women included in the analyses are described in Table S1A. Most of the women were non-smokers, not using HT, and 41.8% were not using any other medication (MED). In average, smokers consumed 2.8 cigarettes (sd 3.8) before blood sampling and 10.2 cigarettes (sd 6.3) the day before. The mean body mass index (BMI) was 25.6 kg/m^2^ (SD 4.2) with most women either of normal BMI (51.7%) or overweight/obese (45.8%). Women in our study range from 48 to 62 years of age (mean 55.7; SD 3.6). Age was significantly associated with smoking (Chi-square *p*-value = 0.01).

#### Data analysis strategy

**Figure 1:**
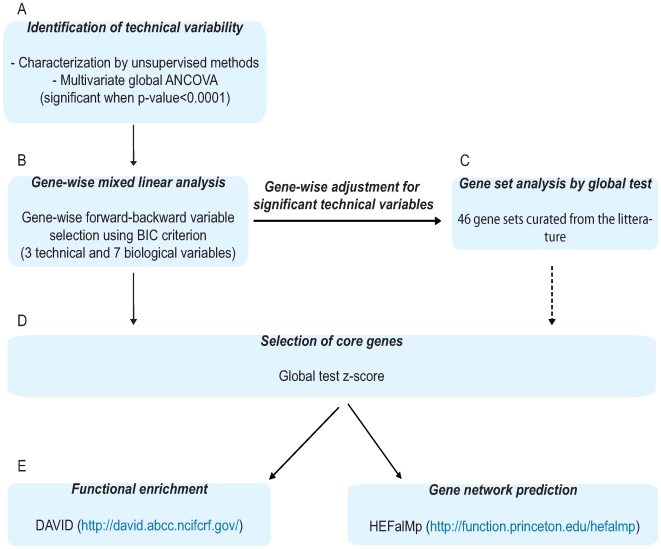
Schematic representation of data analysis.

Using the data analysis strategy outlined in Figure 1, three among eight reported technical variables found significant in multivariate global analysis of covariance (ANCOVA) [25] (Figure 1A), as well as three biological (age, body mass index, fasting status), and three exposure variables (smoking, HT and MED) were included in the forward-backward variable selection by the mixed linear model run for each probe (Figure 1B). Additionally, as an interaction between HT and MED use was significant in the blood expression profiles, we also included an interaction variable to account for this in our model. Since model selection based on Bayesian information criterion (BIC) does not take into account issues of multiple testing, we filtered the gene sets based on the z-score value from global test [26] and set a threshold which maximizes the discovery of true positives (weight = 2) *versus* false positives (weight = 1) associated to each variable (Figure 1D). The z-score obtained from the global test [26] is a useful analytical tool to reduce probes that have previously been found differentially expressed to a core set by estimating the contribution of each probe to the overall measure of association for this set to a specific variable. Throughout this report, we refer to probes filtered by global test z-score more likely to be true positives as core probes. We applied functional clustering via the Database for Annotation, Visualization, and Integrated Discovery (DAVID) [27] (http://david.abcc.ncifcrf.gov/) and gene network predictions via HEFalMp [28] (http://function.princeton.edu/hefalmp) to the resultant core gene list for each phenotype, in order to identify molecular pathways and processes that are variable across our panel of healthy subjects (Figure 1E).

As a complementary approach (Figure 1C), we curated gene sets from published articles focusing on normal variability in blood or gene expression profiles related to exposure, and subsequently conducted gene set enrichment analysis via the global test [26]. In total, 42 such gene sets were identified from 14 published papers and the Kyoto Encyclopedia of Genes and Genomes (KEGG) database [29] (Table S2).

### Investigation of baseline variation in gene expression changes in blood

#### Global analysis of covariance

We investigated the effects of technical variability by searching for associations between the global blood gene expression profiles and eight reported technical variables (Table S1B) that catalog day-to-day RNA processing, RNA/cRNA purity and concentration. All technical variables were highly significant in the univariate global ANCOVA [25] but three variables (i.e. array lot number, RNA extraction date, time between blood collection and freezing) remain significant the multivariate analysis with permuted *p*-value less than 0.0001 (Table S3).

EigenR2 analysis and probe sets variability. Via an eigenR2 analysis [30] which is a high-dimensional version of the classic R2 statistic, we estimated that the three above-mentioned technical variables and the six biological/exposure variables explained 46.5% and 8.1% of the overall variation in gene expression, respectively. These results suggest that the contributions of technical variability result in a level of random noise which is deemed to be high for this large sample set even after standard normalization.

Under gene-wise linear model selected by BIC criterion, each probe was found to be associated with 3.4 variables on average (total 10 variables considered, SD 1.2). As complementary analysis, we considered only those probes that were uniquely associated with a single variable to capture specific signals related to one biological variable. However, since most probes (77%) showed expression patterns that associate with array lot, we did not filter probes from this subsequent analysis if they were associated with this technical variable. Throughout this report, we refer to probes that meet this criterion as biologically uniquely associated with a variable of interest (Table S4).

### Investigation of variation in gene expression changes in blood associated with biological and exposure variables

#### Molecular effects of smoking mirrored in blood

Gene-wise mixed linear analysis identified 3,024 probes related to smoking of which 98.1% are core probes (FDR = 0.01; Table 1). Via DAVID, we identified several biological processes significantly over-represented in the smoking-associated set of genes (Table 2) including enrichments for “rhodopsin-like, G protein coupled receptor activity” (DAVID cluster of 5 biological processes, median FDR = 1.60 10^−6^; Table 2) and “olfactory receptor activity, sensory perception of smell/chemical stimulus” (DAVID cluster of 6 biological processes, median FDR = 0.46%, Table 2). Two sub-endothelial adhesive proteins (fibronectin and thrombospondin, Table 2) were significantly deregulated by smoking. Finally, specific (e.g. monoamine oxidoreductase activity) as well as more general processes (e.g. substrate-specific/ion trans-membrane transporter activity and receptor activity) were significantly enriched in the smoking-associated genes (Table 2). When investigating core genes biologically uniquely associated by smoking (N = 174; Table S4), we identified one consistent significant enrichment in “oxidoreductase activity acting on NAD(P)H” (DAVID cluster of 5 biological processes, median FDR = 2.65%). The genes biologically uniquely up-regulated in non smokers includes *ARHGEF19* encoding a Rho GTPase involved in regulation of small GTPase and signal transduction processes.

**Table 1:** Gene-wise linear analysis conducted for each probe (N = 16185) and global test z-score filtering conducted for gene sets associated to each biological variable.

	Gene-wise linear analysis	Global test z-score filtering
	N probes	N of preselected probes (FDR)
Age class	40	36 (0.01)
Fasting	13,611	269 (0.23)
Body mass index class	3,098	678 (0.20)
Smoking	3,024	2966 (0.01)
Use of medication (MED)	8,636	1302 (0.20)
Hormone therapy use (HT)	5,739	538 (0.21)
Interaction HT*MED	1,807	1245 (0.10)

**Table 2:** Functional enrichment of core probes associated with smoking status in gene-wise mixed linear model based on BIC criterion and filtered based on global test z-score (N = 2966).

Functional cluster	GO terms (N)	Keywords	Genes	Median p-value	Median Fold Enrichment	Median FDR (%)
Group 1	5	Rhodopsin-like, G protein coupled receptor activity	*GPR92, P2RY6, P2RY11, UTS2R, GPR75, GPR35, GNAO1, OR2W3, GNAQ, OPRD1, PLCD3, TBXA2R, OR2B11, GPR56, GNA11, OR8S1, MRGPRD, GPR171, OR1D5, OR10H5, OR4A47, OR51G1, PLCD1, ADRB1*	4.18E-08	1.88	7.02E-05
Group 2	7	Olfactory receptor activity, sensory perception of smell/chemical stimulus	*OR7A17, OR2W3, OR8A1, OR2B1, OR8S1, OR13J1, OR4D1, OR6B2, OR3A2, OR10H1, OR2A14, OR1D5, OR7C2, OR6N1, OR1L8, OR5L1, OR10H5, OR9G1, OR4M1, OR4A47, OR51G1, OR2H2, OR2L2*	2.28E-04	2.13	0.40
Group 3	3	Fibronectin, type III	*NPHS1, TRIM67, IL27RA, SDK1, EGFLAM, IGSF9B, IGF1R, ELFN2, MERTK, IL7R, EPHA4, LRRN3, DSCAM, LOC221091, NOPE, IL12RB2, PHYHIPL, IL4R, ROBO4, IFNAR1, IL11RA, EPHA1, LRFN1*	4.99E-04	2.08	0.94
Group 4	3	Receptor activity, molecular/signal transducer activity	*ASGR1, ITGA10, P2RY6, P2RY11, UTS2R, PTCHD2, GPR75, OR2W3, PRKCG, MED8, OR2B11, TNFRSF25, PRKCZ, TRPV4, KIR2DS4, OR8S1, MRGPRD, FGFR2, GPR171, OR10H5, IGSF10, LILRB4, EPHA1, ILDR1*	8.99E-03	1.16	14.7
Group 5	4	Substrate-specific/ion transmembrane transporter activity	*SLC6A17, KCNMA1, SEC61B, AKAP8, PLLP, KCNF1, SLC6A7, P2RX2, SLC16A8, AQP5, ATP5G3, COX4I1, KCNIP3, IMAA, LSR, SEC61G, FLJ20433, TRPV4, PEA15, KCTD10, SLC6A8*	0.009	1.27	14.6
Group 6	1	Thrombospondin, subtype 1	*SEMA5B, ADAMTS2, SSPO, C8B, ADAMTS10, ADAMTS12, ADAMTS14*	0.007	3.52	12.3
Group 7	1	oxidoreductase activity, acting on paired donors, with incorporation or reduction of molecular oxygen, reduced flavin or flavoprotein as one donor, and incorporation of one atom of oxygen	*CYP2B6, CYP1B1, CYP4F11, CYP4F8, TBXAS1, CYP2B7P1, CYP2D6*	0.007	3.53	11.3

**Figure 2:**
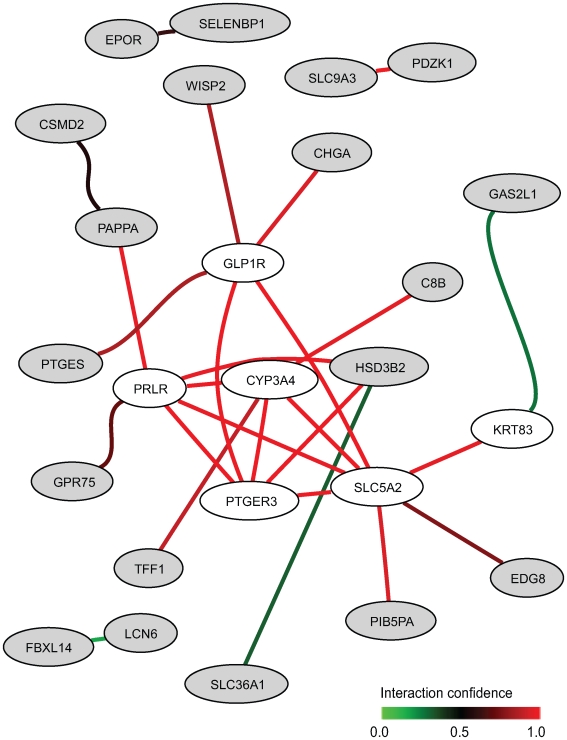
Network between top genes (in grey) in the two hormone-related gene sets up-regulated in non-smokers and genes (in white) predicted by HEFalMp in relation to this query considering all genes in all biological processes.

Seventeen of the 42 curated gene sets were found to be significantly enriched (FDR<0.02) in our dataset with respect to smoking status (Table 3). Two studies [7],[31] have previously investigated exposure effect of smoking on blood gene expression and identified two signatures overlapping by only a single gene. In our dataset, 34 and 19 probes on our microarray could be matched to the 26-gene and 17-gene signatures from [7] and [31], respectively. Both of the gene sets induced the most significant enrichment scores associated with smoking status (Table 3). The comparative *p*-value indicates that only 0.2% and 8.2% respectively of random gene sets of the same size as the two signatures would have a larger standardized test statistic. In the first gene set [7], we identified the 13 core genes constitutive of a gene network predicted by HEFalMp involved in response to wounding, acute inflammatory response and cell chemotaxis (Figure S1). Other curated gene sets were significantly enriched according to smoking status with a FDR <0.02 and comparative *p*-value <0.50. In non-smokers, two gene sets related to growth factor and stress response signaling due to exercise [32] were up-regulated (Table 3). Several signatures of blood cell subtype were significantly enriched with respect to smoking status. In particular, monocyte-specific genes were up-regulated whereas red blood cell- and natural killer cell- specific genes were down-regulated in smokers [18]. The seven-gene signature related to age [13] was enriched with smoking status in our dataset, as were two hormone-related gene sets [10],[33]. Using the core genes of these two hormone-related gene sets up-regulated in non smokers, HEFalMp revealed a gene network (Figure 2) enriched in neuroactive ligand-receptor interactions. It identifies increased expression of sphingosine 1-phosphate receptor (*EDG8*) and predicted interactions of the query core genes with prolactin (*PRLR*), glucagon (*GL1PR*), and prostaglandin E2 (*PTGER3*) receptors (Figure 2).

**Table 3:** Significant gene sets curated from the literature associated with smoking status using the global test.

	Tested genes	*p*-value	FDR adjusted	Comparative p-value	# core genes (probes) up-regulated in smokers	# core genes (probes) up-regulated in non smokers
**Smoking signatures [7];[31]**						
Lampe et al.	26	5.77E-08	1.21E-06	0.002	*IL1B, CYP1B1 ZNF609, EPB41L3, VCAN, DNAJC7, TNNT1*(10)	*AOC2, NRG1*(3)
Van Leeuwen et al.	10	8.27E-04	6.67E-05	0.09	*SERPINB2, IL1B, PCK2, ERCC5, ENO1*(5)	*HAMP, ACO1* (3)
**Exercise signatures [32]**						
Growth factor and transcription	12	1.90E-06	2.66E-05	0.008	*CYP1B1, TCF8* (2)	*CLIC3, GPR56, AKR1C3* (4)
Stress response	10	1.22E-04	6.67E-04	0.09	*HSPA1A* (1)	*SPON2, HSPB1* (2)
**Blood cell subtype signatures [13],[18]**						
Monocytes	17	4.33E-04	0.002	0.23	*FLJ20701, CSPG2, PLA2G7, MARCO, VNN1, IFIT1, CD1D, CD14, RNASE6, MX1, PGD* (11)	*CGI-38* (1)
Red blood cells	33	0.002	0.004	0.45		*CSDA, SELENBP1, MKRN1, EPB42, MAP2K3, BAG1, UBB, FKBP8, GMPR, BNIP3L, BCL2L1, PPM1A, NXPH3, CHI3L2, GSPT2, GSPT1, SNCA* (21)
Natural killer cells in PBMC	17	9.27E-04	0.003	0.35	*CSPG2* (1)	*SPON2, GPR56, MLC1, CX3CR* (4)
**Hormone-related signatures**						
Estrogen-related genes [29],[33]	65	1.06E-04	6.67E-04	0.31	*EPB41L3, ANXA3, RAB31, SRD5A1, SLC39A6* (6)	*PDZK1, TFF1, SELENBP1,HSD3B2, EPOR, WISP2, PIB5PA, PTGES* (8)
Hormone therapy signature [10]	46	4.32E-04	0.002	0.34	*RNF144, CREB5, NME6* (4)	*CSMD2, SLC9A3, SLC36A1, C8B, GPR75, EDG8, CHGA, LCN6,GAS2L1, FBXL14, PAPPA* (12)
**Divers**						
Age signature [13]	9	1.27E-04	6.67E-04	0.08	*IGJ* (1)	*HLA-DQB2* (1)

#### BMI class and mirrored metabolic effect on the blood transcriptome

Among the probes associated with BMI class in the gene-wise linear analysis (N = 3098), 678 were core probes (FDR = 0.20; Table 1). We identified enrichment for several biological processes involved in adaptive immune related responses (Table 4). Of particular note is the identification of a signature for diabetes type I (DAVID cluster of 9 biological processes, median FDR = 5.60 10^−6^; Table 4). In women with normal BMI, two curated gene sets related to inflammatory and stress response signaling due to exercise [32] were up-regulated (Table 5).

**Table 4:** Functional enrichment of core probes associated with BMI class in gene-wise mixed linear model based on BIC criterion and filtered based on global test z-score (N = 678).

Functional cluster	GO terms (N)	Keywords	Genes	Median p-value	Median Fold Enrichment	Median FDR (%)
Group 1	5	Immunoglobulin/major histocompatibility complex motif, Immunoglobulin C1-set	*HLA-DMB, CD1C, CTSE, HLA-DPA1, HLA-DMA, HLA-DOA, HLA-DQA2, IGKC, HLA-DPB1, IGHG2, HLA-DRA, HLA-DRB5, HLA-C, HLA-DRB5, IGHM, IGHD, HLA-B*	9.92E-08	6.89	1.51E-04
Group 2	9	Type I diabetes mellitus, MHC class II	*GZMB, HLA-DMB, CTSE, HLA-DPA1, HLA-DMA, HLA-DOA, LTA, HLA-DQA2, HLA-DPB1, HLA-DRA, HLA-DRB5, HLA-C, HLA-DRB5, HLA-B*	1.78E-07	14.2	3.11E-04
Group 3	4	MHC class II, alpha chain	*HLA-DPA1, HLA-DMA, HLA-DOA, HLA-DQA2, HLA-DPB1, HLA-DRA, HLA-DRB5, HLA-C, HLA-DRB5, HLA-B*	8.44E-05	13.4	1.71E-01
Group 4	5	positive regulation of immune system process	*IL15, HLA-DMA, CD40, UBASH3A, CD46, TRAF2, CD55, IGHM, SMAD3, KRT1, FCER1A*	0.001	3.81	1.12
Group 5	3	Immunoglobulin E binding	*MS4A2, FCER2, LGALS3, FCER1A*	0.001	16.1	1.80
Group 6	7	Lymphocyte B/immunoglobulin mediated immune response, adaptive immune response	*HLA-DMA, CD40, CD46, CD55, IGHM, TNFSF13, TRAF2, FCER1A, IL15, KRT1*	0.008	3.41	13.86
Group 7	2	Immunoglobulin C region	*IGKC, IGHG2, IGHM, IGHD, TRA@*	0.006	10.8	9.94

**Table 5:** Significant gene sets curated from the literature associated with BMI class using global test.

	Tested genes	*p*-value	FDR adjusted	Comparative p-value	Core genes up-regulated in women at normal BMI (N probes)	Core genes up-regulated in overweighted women (N probes)
**Exercise signatures [32]**						
Inflammatory response	18	0.0005	0.02	<0.0001	*GZMB, XCL1, PTGDS, GNLY, NCR3, XCL2, CST7, CCL4, GZMA, CTSW* (10)	*CD22*(1)
Stress response	10	0.0009	0.02	0.004	*DUSP2, DUSP1, HIF1A* (3)	

#### Genes related to fasting status, medication, and hormone therapy use: correlation and interaction of complex signals

The biological variables fasting, MED, and HT use induced the most significant probes under our gene-wise mixed linear models (84.1%, 62.7% and 44.5% of all genes, respectively; Table 1). In fact, there was a high degree of overlap between all three variables (40.0%, 5,775 genes in total), and 74.5% are associated with at least two variables.

As noted earlier, an interaction between HT and MED in relation to the blood gene expression profiles was statistically significant (permuted *p*-value = 0.03). HT use was associated with the blood gene expression profiles with a multivariate permuted *p*-value of 0.09 and 0.44 in users and non-users of other medications, respectively. MED was associated with the blood gene expression profiles with a multivariate permuted *p*-value of 0.06 and 0.38 in non-users and users of HT, respectively. Further analyses are required in order to investigate the different categories of MED, HT regimens informed by questionnaire and hormone levels measured in plasma, as well as their interactions in relation to blood gene expression profiles.

Of the 13611 probes identified as related to fasting (Table 1), 269 were identified as core probes (FDR = 0.23). This latter probe list was significantly enriched in regulation of transcription and RNA metabolic process (DAVID cluster of 11 biological processes; median FDR = 8.30%, Table 6) partly involving deregulation of zinc finger proteins (DAVID cluster of 3 biological processes; median FDR = 0.65%, Table 6) or bromo-domain containing proteins (DAVID cluster of 1 biological process; median FDR = 7.7%, Table 6) involved in chromatin modification. In accordance with these results, the core probes (N = 36, Table S4) biologically uniquely associated with fasting women were significantly enriched in chromatin modification and control of gene expression by vitamin D receptor (DAVID cluster of 2 biological processes; median FDR = 10.3%).

**Table 6:** Functional enrichment of core probes associated with fasting status in gene-wise mixed linear model based on BIC criterion and filtered based on global test z-score (N = 269).

Functional cluster	GO terms (N)	Keywords	Genes	Median p-value	Median Fold Enrichment	Median FDR (%)
Group 1	13	Regulation of transcription, cellular metabolic process, RNA metabolic process, nucleobase, nucleoside, nucleotide and nucleic acid metabolic process	*ACAD8, MYCBP2, MED29, MTA2, EID2, ZNF182, LIMD1, RBM9, BAZ2A, LOC344167, SLC6A3, SUDS3, ZNF395, BRD7, ZNF555, POGZ, ZNF282, ATF7IP, PBXIP1, ZKSCAN2, ZNF324, ZNF740, CEBPE, KHSRP*	0.001	1.60	1.26
Group 2	1	Zinc finger C2H2 type 2	*KLF13, POGZ, PRDM2, RLF, ZNF264, ZNF282, ZNF333, ZNF345, ZNF396, ZNF585A*	1.05E-02	2.55	19.66
Group 3	1	Bromo domain	*SMARCA2, SMARCA4, CREBBP, BAZ1B*	0.009	9.0	17.1

Finally, none of the 14 gene sets curated from the literature were significantly enriched in our dataset with respect to fasting status. A similar absence of significant enrichment was observed for a list of 1356 genes associated with fasting in peripheral blood mononuclear cells [34], of which 784 probes were identifiable in our dataset.

#### Age difference in postmenopausal women and its weak effect on blood gene expression profiles

No significant enrichment of biological processes was observed for the 40 probes including 36 core probes associated with age group (FDR = 0.01; Table 1). With respect to gene set enrichment analysis, the immunoglobulin gene set (N = 36) had the lowest global test enrichment *p*-value (*p*-value = 0.03), but a high false discovery rate (FDR = 0.92). One publication [13] found a gene list (N = 14 genes; N = 9 after mapping to our Applied Biosystems probe IDs) derived from blood and associated with age but was not significantly enriched in our dataset (global test *p*-value = 0.45).

## Discussion

Peripheral blood is an ideal surrogate tissue as it has the potential to reflect responses to changes in the immediate and distant environments by alterations of gene expression levels. Given the number of factors that influence gene regulation and expression, it is not surprising that often more than one strong signal is present in any given high-dimensional dataset. The external validity of NOWAC as a representative sample of the Norwegian female population has been verified in several methodological analyses and found to be acceptable [35]. Studies of the internal validity, including reliability, have been undertaken for dietary questions [36],[37], menopausal status, and use of HT [36],[38], whereas validation of variables measuring physical activity remain ongoing.

### Inter-individual variability

In addition to technical variability, substantial differences in gene expression profiles were identified between individuals with respect to exposure. Overall, the functional enrichment of significant single genes and gene set enrichment analyses show that high-throughput gene expression studies implicate similar (although not identical) underlying biology across several studies. Whereas age did not induce a large effect in blood gene expression for our cohort of postmenopausal women aged from 48 to 62 years, pathways and gene sets affected by smoking and, to a lesser extent both BMI and fasting, are numerous and interconnected. Some expression profiles associated with these variables may also be associated with other factors (e.g., lower levels of exercise, age). A host of new candidate genes for regulation by inter-individual (fasting, BMI) and exposure (smoking) factors were identified which could be used as a basis for hypothesis development.

Several processes associated with smoking were involved in cardiovascular regulation by G-coupled receptors (i.e. purinergic, adrenergic beta-1, urotensin II or thromboxan A2 receptors) or protein activity (i.e. thrombospondin type-1, fibronectin type-3). Consistent with previous observations that smoking reduces olfactory sensitivity in a dose- and time-dependent manner [39],[40], we find that smoking significantly impairs blood gene expression of olfactory receptors. We also observed that smokers have deregulated gene expressions of several P450 cytochromes which catalyse mono-oxygenase activity that can both toxify and detoxify carcinogenic compounds. As established in normal lung [41] and rats [42], smokers tend to have a small increase in NAD(P)H:(quinone-acceptor) oxidoreductase compared to non-smokers.

Two previous studies [7],[31] have examined the effects of cigarette smoking on leukocyte gene expression in circulation and both of the associated signatures had the most significant enrichment scores over all gene sets considered here. Inflammatory responses previously associated with smoking [7] were up-regulated in the blood expression of smokers in our dataset. Lending support that smoking has immune and inflammatory effects, specific blood cell gene signatures [13],[18] (i.e. increased monocytes and decreased red blood cell and natural killer cell signalling) were differentially expressed according to smoking status. This is consistent with previous observations showing that the total numbers of peripheral leukocytes differ by smoking status [43],[44]. Core genes up-regulated in non-smokers from the enriched hormone-related gene sets [10],[33] were predicted to be involved in neuroactive ligand-receptor interactions like prostaglandin receptors. Elevated prostaglandin E2 synthesis has been previously reported in smokers in comparison with non-smokers [45],[46]. The predicted gene network also reflects the effect of smoking on hormone levels with increased secretion of prolactin and glucagon [47]. Two pathways related to exercise [32] were also found up-regulated in non-smokers, which may simply be due to an underlying prevalence of active exercisers in non-smokers [48].

In our study, we found BMI class associated with blood gene expression changes involved in several immune processes including diabetes type I. It has been reported that several immune functions are dysregulated in obesity [49],[50] and both genetic and environmental factors such as obesity have been implicated as triggers in the pathogenesis of diabetes. The role of autoimmunity in the origins of type I diabetes is well-known, including a role in latent autoimmune diabetes in adults [51] and several observations suggest that autoimmunity may be part of type II diabetes [52]–[55]. Finally, two pathways related to exercise [32] were also up-regulated in women with normal BMI which may be due to a higher prevalence of physical exercise than in overweight/obese women.

Of all the variables considered, fasting was associated with the largest number of genes, but few genes were identified as core genes possibly due to the limited number of fasting women (N = 28) at the time of blood sampling. Selection of core genes aims to select a subset of true positives which work together (possibly in similar pathways) towards significance of the set. The significant core genes associated with fasting were generally involved in gene expression regulation and chromatin modification [56]–[58]. Much of our understanding of the effects of nutrition on chromatin structure has been gleaned from model organisms, especially *S. cerevisiae*, *C. elegans*, *Drosophila*, and mice [59]. In humans, two previous studies were unable to characterize acute effects of food intake in blood gene expression profiles [13],[18]. One putative 784-gene signature exists [34], however only 49 genes associated with fasting overlap with this signature. This may simply be due to chance.

Due to a significant interaction between HT and MED within our profiles, further analyses with a larger sample size are needed in order to investigate the different categories of medications, HT regimens and hormone levels, as well as their interactions in blood.

### Consistency with other studies

Differences between the genes identified and the interpretation of results in the various studies discussed here are likely to have resulted from technical differences in the array platforms used, the subset of blood cells analyzed, and the chosen analytical procedures. Several studies [12]–[18] examined how gene expression profiles of blood samples are affected by technical variables. Specific blood sample collection methods result in the isolation of different blood cell subpopulations. White blood cells have been defined as the most transcriptionally active of all cell types in blood and may give the most sensitive gene expression profiles in response to defined factors [60]. In large epidemiological studies, RNA stabilization is compulsory and PAXgene tubes have been found satisfactory to stabilize and enable RNA extraction from whole blood cells [61]. While high proportions of globin RNA could reduce sensitivity with respect to certain microarray platforms [60],[62],[63], we previously investigated two globin reduction protocols and determined that they were not beneficial when Applied Biosystems (AB) microarrays are used [23]. We found that RNA extraction and one variable related to RNA degradation (i.e. time between blood collection and freezing) had a significant global effect on blood gene expression profiles. In addition to normalization preprocessing, our results suggest that technical variability should not be ignored and possible adjustment for technical sources of variability should be considered in any analysis. Techniques such as surrogate variable analysis [64] may adjust for hidden sources of heterogeneity and large-scale dependence in gene expression studies [65]. As an example in our study, 25 significant surrogate variables were highly correlated to the strongest identified technical sources of noise, array lot number (canonical correlation r^2^ = 0.95), time between blood collection and freezing (canonical correlation r^2^ = 0.62) and RNA extraction (canonical correlation r^2^ = 0.43).

After adjustment for technical variability, our analysis demonstrates the ability to find significant similarities between studies by focusing on the biological implications of the gene sets from each individual study, rather than the specific single genes that met the criteria for significant differential expression in each individual study. They lend support to the idea that blood gene expression studies can indeed detect exposure-specific differences and that failure to consider this type of biological variation can result in the misidentification of genes when investigating predictive, diagnostic or prognostic signatures in blood.

In conclusion, this study extends the limited baseline information currently available that describes normal patterns of variation in blood gene expression. The data generated have been made freely available and should represent a useful resource for the design of future studies including power calculations. Our results confirm the feasibility of identifying signatures of inter-individual factors (e.g. fasting, BMI) and exposure factors (e.g. smoking) in blood-based gene expression profiles, and reinforces the need for proper study design, sample preparation, and technical analysis.

## Methods

### Ethics statement

We have received approval from the Regional Committee for Medical Research Ethics for the collection and storing of questionnaire information and blood samples. The informed consent formula explicitly mentions that the blood samples can be used for gene expression analyses as well as large-scale genotyping.

All data are stored and handled according to the permission given by the Norwegian Data Inspectorate. The Directorate of Health and Social affairs (SHD) has given us an exemption from the confidentiality of information in national registers.

Before use of the biological material, a request has been sent to the regional ethical committee for Northern-Norway. Use of biological material requires permission according to laws pertaining to biotechnology and gene technology, both of which are administered by the SHD.

### Subjects

The women are part of the Norwegian Women and Cancer (NOWAC) study (http://uit.no/kk/NOWAC/) consisting of 172471 women who were 30 to 70 years of age at recruitment from 1991 to 2006 [22]. The NOWAC postgenome cohort study [21] consists of approximately 50,000 women born between 1943 and 1957, randomly drawn in groups of 500 from the NOWAC registers, who gave blood samples between 2003 and 2006 and filled in a two-page questionnaire. The two-page questionnaire included questions regarding menopausal status, weight, height; past week exposure to smoking, HT, oral contraceptives, other MED, omega-3 fatty acid, soy or other dietary supplements; and details concerning blood specimen collection (date, hour, posture). Women included in the present study received a blood collection kit and an accompanying two-page questionnaire by mail in April 2005. Among the group of 500 women, 444 (89%) returned both citrate and PAXgene blood RNA (PreAnalytiX GmbH, Hembrechtikon, Switzerland) tubes; 3.3% declined to participate, 0.7% had died or migrated and 7% did not respond. Samples were included in the study according to the following inclusion criteria: the donor was postmenopausal (99 donors excluded), blood was successfully collected in one PAXgene tube and in two plasma collection tubes (8 donors excluded), and the samples were frozen within 3 days from blood collection (9 donors excluded). Based on these criteria, 328 PAXgene blood samples were included for RNA extraction.

### RNA isolation and quality control

PAXgene blood RNA tubes were thawed at room temperature for 4 h. 500 µL of blood was removed and stored on −70°C for future use. Total RNA was isolated using the PAXgene Blood RNA Isolation Kit, according to the manufacturer's manual. RNA quantity and purity was assessed using the NanoDrop ND-1000 spectrophotometer (ThermoFisher Scientific, Wilmington, Delaware, USA). The absorbance ratio of 260 nm and 280 nm (A260/A280) was between 1.93 and 2.1 for all samples included for further analysis. The Experion automated electrophoresis system (BioRad, Hercules, CA, USA) and the RNA StdSens Analysis Kit was used to evaluate RNA integrity of a randomized 32% of the samples, according to the instruction manual. The electropherograms were inspected for clear ribosomal peaks. We were not able to analyze any numerical criteria corresponding to electrophoresis patterns, because this information was not available. Thirty nine samples were excluded from further analysis due to insufficient RNA purity, yield or integrity. RNA samples were kept at −70°C until further use.

### Microarray-based profiling and image analysis

After exclusion based on study design and RNA quality and quantity criteria, samples were analyzed using the Applied Biosystems (AB) expression array system (Foster City, Lousiana, USA). 500 ng total RNA was used for amplification by the NanoAmp RT-IVT labeling kit from AB for one round of amplification, in accordance with the manufacturer's manual. Briefly, the 1^st^ strand of cDNA was synthesized by reverse transcription using the T7-oligo (dT) primer, followed by 2^nd^ strand synthesis. The double-stranded cDNA was purified, and used as template for *in vitro* transcription (IVT). During IVT, digoxigenin (DIG)-labeled UTP was incorporated into the cRNA. The quantity and purity of the cRNA was measured on the NanoDrop ND-1000, and the cRNA was stored on −70°C until further use. 10 µg of DIG-labeled cRNA was fragmented and hybridized to AB Human Genome Survey Microarray V2.0, in accordance with the Chemiluminescence Detection Kit Protocol.

The AB Human Genome Survey Microarray V2.0 contains 277 control probes and 32,878 probes for the interrogation of 29,098 genes. AB Expression System software was used to extract signal intensities, signal to noise ratios (S/N) and flagging.

### Data analysis

A total of 304 arrays including 15 technical replicates were analyzed. Data analysis was performed using R (http://cran.r-project.org), an open-source-interpreted computer language for statistical computation and graphics, and tools from the Bioconductor project (http://www.bioconductor.org), adapted to our needs. Using R, we set the expression intensity to “missing” for genes with flagging value >8191 (threshold recommended by the microarray manufacturer). For a set of technical replicate arrays from the same subject, we excluded the array with the least number of probes that had a S/N exceeding 3. Furthermore, arrays (N = 3) where less than 40% of the probes had a S/N≥3 were also removed from the analysis. Individual probes were not considered, if the S/N exceeded 3 in less than 50% of the samples. After sample and probe filtration, we proceeded with a log2 transformation, quantile normalization and imputation of missing values using 10-nearest neighbourhood method [66]. A total of 286 arrays and 16185 probes are analyzed. Microarray data have been deposited at Gene Expression Omnibus (GEO; http://www.ncbi.nlm.nih.gov/geo) accession number GSE15289.

The global ANCOVA [25] was carried out by comparison of linear models via the extra sum of squares principle to test for the univariate and multivariate association between global expression values and technical variables. All significant technical variables with a permuted *p*-value <0.001 identified in the ANCOVA multivariate analysis were included in the gene-wise linear model selection as random (array lot number, RNA extraction date) and fixed (time between blood collection and freezing) variables.

Forward-backward variable selection was used to select gene-wise model based on BIC. Linear mixed models were used to test the association of each gene with the significant technical and all biological variables. The z-score from the global test [26] was used to select core probes that most strongly explain the difference between groups setting a FDR [67] threshold which maximizes the discovery of true positives (weight = 2) *versus* false positives (weight = 1) associated with each variable. Gene set enrichment analysis was conducted using the global test [26], which offers the opportunity to compare two or more groups while taking into account the association between probe sets as well as their individual effects. When testing several gene sets curated from the literature, we adjusted for multiple testing using FDR [67]. Functional clustering and gene networks prediction were performed with the Database for Annotation, Visualization, and Integrated Discovery (DAVID) at http://david.abcc.ncifcrf.gov/ [27], and the Human Experimental/Functional Mapper (HEFalMp) [28] at http://function.princeton.edu/hefalmp, respectively.

## Supporting Information

Figure S1 Network between core genes (in grey) related to smoking in the gene set identified by Lampe et al. [7] and genes (in white) predicted by Hefalmp in relation with this query considering all genes in all biological processes.
(1.17 MB EPS)

Table S1 Characteristics of (A) women included in the analysis and (B) blood sample processing.
(0.06 MB DOC)

Table S2 Gene sets curated from literature.
(0.13 MB DOC)

Table S3 Univariate and multivariate global ANCOVA analysis investigating technical variables.
(0.03 MB DOC)

Table S4 Gene-wise linear analysis conducted for each probe (N = 16,185) and global test z-score filtering conducted for gene sets biologically uniquely associated to each biological variable.
(0.03 MB DOC)
