# Supplementary material for: Deciphering Normal Blood Gene Expression Variation—The NOWAC Postgenome Study
Source: PLoS Genet. 2010 Mar 12;6(3):e1000873. doi: 10.1371/journal.pgen.1000873 (PMC2837385; doi:10.1371/journal.pgen.1000873)
Supplement: Table S1 — Characteristics of (A) women included in the analysis and (B) blood sample processing. (0.06 MB DOC) [file pgen.1000873.s002.doc]

**Table S1.** Characteristics of (A) women included in the analysis and (B) blood sample processing

1. **(B)**

|  |  | **Duration** | **Levels** |
| --- | --- | --- | --- |
| **Laboratory work** | **RNA extraction date** | 10 months | 50 days |
|  | **RNA amplification date** | 18 months | 34 days |
|  | **Array lot number** |  | 30 levels |
|  |  | **Mean** | **sd** |
| **Blood samples** | **RNA concentration (ng/µL)** | 95.4 | 45.8 |
|  | **RNA 260/280 ratio** | 2.03 | 0.02 |
|  | **RNA 260/230 ratio** | 1.57 | 0.41 |
|  | **cRNA concentration (ng/µL)** | 429.4 | 171.1 |
|  | **cRNA 260/280 ratio** | 2.15 | 0.04 |
|  | **cRNA 260/230 ratio** | 1.64 | 0.35 |
|  |  | **N** | **Percent** |
| **Blood collection** | **Time between blood collection and storage (days)**  1  2  3 | 197  72  17 | **68.9**  25.2  5.9 |

|  | **Mean** | **sd** |
| --- | --- | --- |
| **Age (years)** | 55.7 | 3.6 |
| **BMI (kg/m^2^)** | 25.6 | 4.2 |
|  | **N** | **Percent** |
| **Hormone therapy use** |  |  |
| Yes | 52 | 18.1 |
| No | 230 | **80.5** |
| Missing | 4 | 1.4 |
| **Smoking status** |  |  |
| Non smoker | 211 | **73.9** |
| Smoker | 74 | 25.8 |
| Missing | 1 | 0.3 |
| **Medication use** |  |  |
| Yes | 161 | **56.5** |
| No | 120 | 41.8 |
| Missing | 5 | 1.7 |
| **Fasting blood samples** |  |  |
| Yes | 28 | 9.7 |
| No | 250 | **87.5** |
| Missing | 8 | 2.8 |
