# Supplementary material for: Deciphering Normal Blood Gene Expression Variation—The NOWAC Postgenome Study
Source: PLoS Genet. 2010 Mar 12;6(3):e1000873. doi: 10.1371/journal.pgen.1000873 (PMC2837385; doi:10.1371/journal.pgen.1000873)
Supplement: Table S2 — Gene sets curated from literature. (0.13 MB DOC) [file pgen.1000873.s003.doc]

**Table S2.** Gene sets curated from literature

| **Reference** | **RNA source** | **Expression profiling method** | **Gene sets** | **N genes** |  |
| --- | --- | --- | --- | --- | --- |
| [1] | Whole blood | 5' Nuclease PCR assay | Interleukins | 15 |  |
|  |  |  | Proto-oncogenes | 7 |  |
|  |  |  | General cytokines | 11 |  |
|  |  |  | CD markers | 20 |  |
| [2] | MCF-7 | Affymetrix Hu95A GeneChips | Estrogen deregulated genes | 21 | (+28 hormone related genes from KEGG [3]) |
|  |  |  | Estrogen up-regulated genes | 86 | (+28 hormone related genes from KEGG [3]) |
| [4] | PBMC and whole blood | Standford cDNA microarray | Lymphocyte signature | 42 |  |
|  |  |  | Monocyte signature | 24 |  |
|  |  |  | PBMC signature | 65 |  |
|  |  |  | Neutrophils signature | 31 |  |
|  |  |  | Red blood cell signature | 37 |  |
|  |  |  | Reticulocyte signature | 9 |  |
|  |  |  | Red blood cell and reticulocyte signature | 46 |  |
| [5] | PBMC | Affymetrix HU133A GeneChip. | Stress response associated to exercise | 12 |  |
|  |  |  | Growth factor and transcription factor associated to exercise | 23 |  |
|  |  |  | Inflammatory response to exercise | 24 |  |
| [6] | Neutrophils | Affymetrix HG-U95Av2 GeneChip | Immature *vs* mature neutrophils | 25 |  |
| [7] | Leukocytes | Agilent Hu25K microarray | Individual specific genes | 51 |  |
| [8] | Whole blood | Affymetrix U133A or U133 Plus GeneChip | T cell enriched *vs* leukocytes | 104 |  |
|  |  |  | Monocyte enriched *vs* leukocytes | 86 |  |
|  |  |  | Upregulated genes associated to trauma | 90 |  |
|  |  |  | Downregulated genes associated to trauma | 94 |  |
| [9] | Whole blood | Agilent Hu25k microarray | Genes correlated to cotinine levels | 36 |  |
|  |  |  | Positive correlation with cotinine levels | 27 |  |
|  |  |  | Negative correlation with cotinine levels | 9 |  |
| [10] | PBMC | In house oligomicroarray | Genes related to age | 14 |  |
|  |  |  | BMI-specific genes | 3 |  |
|  |  |  | Natural killer cells in PBMC signature | 26 |  |
|  |  |  | Monocytes in PBMC signature | 50 |  |
| [11] | Whole blood | Agilent 22k oligoarray | Hormone therapy associated to hormone therapy use | 84 |  |
| [12] | Leukocytes | Affymetrix U133A GeneChip | Genes associated to high protein high carbohydrate breakfasts | 28 |  |
|  |  |  | Protein synthesis genes associated to high protein breakfast | 35 |  |
|  |  |  | High interindividual variabililty genes | 20 |  |
| [13] | Whole blood | Phase1 Human Tox 600 cDNA microarray | Genes related to smoking | 17 |  |
|  |  |  |  |  |  |
| **Table S3 (continued)** | | | |  |  |
|  | | | |  |  |
| **Reference** | **RNA source** | **Expression profiling method** | **Gene sets** | **N genes** |  |
| [14] | PBMC | Affymetrix U133 plus 2.0 GeneChip | Genes related to fasting | 1356 |  |
| [15] | Lymphocytes | In-house microarray (Visvikis-Siest et al., 2007) | Drug metabolizing enzymes | 15 |  |
|  |  |  | Transcription factors | 13 |  |
|  |  |  | Transcription factors and drug metabolizing enzymes | 28 |  |
| KEGG [3] |  |  | Estrogen-androgen metabolism | 18 |  |
|  |  |  | Steroid hormone metabolism | 10 |  |
|  |  |  | T cell receptor signalling | 94 |  |
| Other |  |  | Immunoglobulin gene set | 51 |  |

Reference List

1. Tanner MA, Berk LS, Felten DL, Blidy AD, Bit SL, Ruff DW (2002) Substantial changes in gene expression level due to the storage temperature and storage duration of human whole blood. Clin Lab Haematol 24: 337-341.

2. Frasor J, Stossi F, Danes JM, Komm B, Lyttle CR, Katzenellenbogen BS (2004) Selective estrogen receptor modulators: discrimination of agonistic versus antagonistic activities by gene expression profiling in breast cancer cells. Cancer Res 64: 1522-1533.

3. Kanehisa M, Goto S (2000) KEGG: kyoto encyclopedia of genes and genomes. Nucleic Acids Res 28: 27-30.

4. Whitney AR, Diehn M, Popper SJ, Alizadeh AA, Boldrick JC, Relman DA, Brown PO (2003) Individuality and variation in gene expression patterns in human blood. Proc Natl Acad Sci U S A 100: 1896-1901.

5. Connolly PH, Caiozzo VJ, Zaldivar F, Nemet D, Larson J, Hung SP, Heck JD, Hatfield GW, Cooper DM (2004) Effects of exercise on gene expression in human peripheral blood mononuclear cells. J Appl Physiol 97: 1461-1469.

6. Martinelli S, Urosevic M, Daryadel A, Oberholzer PA, Baumann C, Fey MF, Dummer R, Simon HU, Yousefi S (2004) Induction of genes mediating interferon-dependent extracellular trap formation during neutrophil differentiation. J Biol Chem 279: 44123-44132.

7. Radich JP, Mao M, Stepaniants S, Biery M, Castle J, Ward T, Schimmack G, Kobayashi S, Carleton M, Lampe J, Linsley PS (2004) Individual-specific variation of gene expression in peripheral blood leukocytes. Genomics 83: 980-988.

8. Cobb JP, Mindrinos MN, Miller-Graziano C, Calvano SE, Baker HV, Xiao W, Laudanski K, Brownstein BH, Elson CM, Hayden DL, Herndon DN, Lowry SF, Maier RV, Schoenfeld DA, Moldawer LL, Davis RW, Tompkins RG, Baker HV, Bankey P, Billiar T, Brownstein BH, Calvano SE, Camp D, Chaudry I, Cobb JP, Davis RW, Elson CM, Freeman B, Gamelli R, Gibran N, Harbrecht B, Hayden DL, Heagy W, Heimbach D, Herndon DN, Horton J, Hunt J, Laudanski K, Lederer J, Lowry SF, Maier RV, Mannick J, McKinley B, Miller-Graziano C, Mindrinos MN, Minei J, Moldawer LL, Moore E, Moore F, Munford R, Nathens A, O'keefe G, Purdue G, Rahme L, Remick D, Sailors M, Schoenfeld DA, Shapiro M, Silver G, Smith R, Stephanopoulos G, Stormo G, Tompkins RG, Toner M, Warren S, West M, Wolfe S, Xiao W, Young V (2005) Application of genome-wide expression analysis to human health and disease. Proc Natl Acad Sci U S A 102: 4801-4806.

9. Lampe JW, Stepaniants SB, Mao M, Radich JP, Dai H, Linsley PS, Friend SH, Potter JD (2004) Signatures of environmental exposures using peripheral leukocyte gene expression: tobacco smoke. Cancer Epidemiol Biomarkers Prev 13: 445-453.

10. Eady JJ, Wortley GM, Wormstone YM, Hughes JC, Astley SB, Foxall RJ, Doleman JF, Elliott RM (2005) Variation in gene expression profiles of peripheral blood mononuclear cells from healthy volunteers. Physiol Genomics 22: 402-411.

11. Dumeaux V, Johansen J, Borresen-Dale AL, Lund E (2006) Gene expression profiling of whole-blood samples from women exposed to hormone replacement therapy. Mol Cancer Ther 5: 868-876.

12. van Erk MJ, Blom WA, van OB, Hendriks HF (2006) High-protein and high-carbohydrate breakfasts differentially change the transcriptome of human blood cells. Am J Clin Nutr 84: 1233-1241.

13. van Leeuwen DM, van AE, Gottschalk RW, Vlietinck R, Gielen M, van Herwijnen MH, Maas LM, Kleinjans JC, van Delft JH (2007) Cigarette smoke-induced differential gene expression in blood cells from monozygotic twin pairs. Carcinogenesis 28: 691-697.

14. Bouwens M, Afman LA, Muller M (2007) Fasting induces changes in peripheral blood mononuclear cell gene expression profiles related to increases in fatty acid beta-oxidation: functional role of peroxisome proliferator activated receptor alpha in human peripheral blood mononuclear cells. Am J Clin Nutr 86: 1515-1523.

15. Siest G, Jeannesson E, Marteau JB, Samara A, Marie B, Pfister M, Visvikis-Siest S (2008) Transcription factor and drug-metabolizing enzyme gene expression in lymphocytes from healthy human subjects. Drug Metab Dispos 36: 182-189.
