# Supplementary material for: Deciphering Normal Blood Gene Expression Variation—The NOWAC Postgenome Study
Source: PLoS Genet. 2010 Mar 12;6(3):e1000873. doi: 10.1371/journal.pgen.1000873 (PMC2837385; doi:10.1371/journal.pgen.1000873)
Supplement: Table S3 — Univariate and multivariate global ANCOVA analysis investigating technical variables. (0.03 MB DOC) [file pgen.1000873.s004.doc]

**Table S3.** Univariate and multivariate global ANCOVA analysis investigating technical variables.

|  | **Univariate Global Ancova** | **Multivariate* Global Ancova** |
| --- | --- | --- |
|  | **F value (permuted *p*-value)** | **F value (permuted *p*-value)** |
| **Technical variables** |  |  |
| Array lot number | 3.32 (< 0.001) | 2.60 (< 0.001) |
| Amplification date | 2.93 (< 0.001) | - |
| cRNA 260/280 | 4.12 (< 0.001) | 1.92 (0.01) |
| cRNA 260/230 | 2.03 (0.01) | 1.36 (0.06) |
| RNA extraction date | 1.45 (< 0.001) | 1.26 (< 0.001) |
| RNA 260/280 | 2.55 (< 0.001) | 1.27 (0.11) |
| RNA 260/230 | 2.81 (< 0.001) | 1.41 (0.06) |
| Time between blood collection and storage | 5.34 (< 0.001) | 4.50 (< 0.001) |

* Including all significant variables in the univariate analyses. Amplification date was highly correlated to array lot number, so only array lot number was included in the analyses.
