# Supplementary material for: Deciphering Normal Blood Gene Expression Variation—The NOWAC Postgenome Study
Source: PLoS Genet. 2010 Mar 12;6(3):e1000873. doi: 10.1371/journal.pgen.1000873 (PMC2837385; doi:10.1371/journal.pgen.1000873)
Supplement: Table S4 — Gene-wise linear analysis conducted for each probe (N = 16,185) and global test z-score filtering conducted for gene sets biologically uniquely associated to each biological variable. (0.03 MB DOC) [file pgen.1000873.s005.doc]

**Table S4.** Gene-wise linear analysis conducted for each probe (N=16,185) and global test z-score filtering conducted for gene sets biologically uniquely associated to each biological variable

|  | **Gene-wise linear analysis** | **Global test z-score filtering** |
| --- | --- | --- |
|  | **N probes** | **N preselected probes (FDR)** |
| Age class | 6 | 5 (0.0005) |
| Fasting | 1155 | 36 (0.21) |
| Body mass index class | 43 | 20 (0.21) |
| Smoking | 181 | 174 (0.03) |
| Use of medication (MED) | 207 | 98 (0.15) |
| Hormone therapy use (HT) | 78 | 26 (0.16) |
| Interaction HT*MED | 4 | 2 (0.20) |
